# Supplementary material for: HIVprotI: an integrated web based platform for prediction and design of HIV proteins inhibitors
Source: J Cheminform. 2018 Mar 9;10:12. doi: 10.1186/s13321-018-0266-y (PMC5845081; doi:10.1186/s13321-018-0266-y)
Supplement: Supplementary file 2 — Additional file 2. Source code of HIVProtI web server. [file 13321_2018_266_MOESM2_ESM.zip › HIVprotI_Source-code/hivprotI/weka-3-6-6/doc/weka/core/stemmers/Stemmer.html]

Stemmer


---


|  |  |  |
| --- | --- | --- |
| **Overview** \| **Package** \| **Class** \| **Tree** \| **Deprecated** \| **Index** \| **Help** \| **Weka's home** | |  |
| **PREV CLASS**   **NEXT CLASS** | **FRAMES**    **NO FRAMES**     **All Classes** |  |
| SUMMARY: NESTED \| FIELD \| CONSTR \| METHOD | DETAIL: FIELD \| CONSTR \| METHOD |  |


---


## weka.core.stemmers Interface Stemmer

**All Superinterfaces:**: RevisionHandler, java.io.Serializable

**All Known Implementing Classes:**: IteratedLovinsStemmer, LovinsStemmer, NullStemmer, SnowballStemmer

---

``` public interface Stemmer extends java.io.Serializable, RevisionHandler ```

Interface for all stemming algorithms.

**Version:**
:   $Revision: 1.3 $

**Author:**
:   FracPete (fracpete at waikato dot ac dot nz)

---

| **Method Summary** | |
| --- | --- |
| `java.lang.String` | `stem(java.lang.String word)`             Stems the given word and returns the stemmed version |

**Methods inherited from interface weka.core.RevisionHandler**

`getRevision`

**Method Detail**

### stem

```
java.lang.String stem(java.lang.String word)
```

:   Stems the given word and returns the stemmed version

    :   **Parameters:**: `word` - the unstemmed word **Returns:**: the stemmed word


---


|  |  |  |
| --- | --- | --- |
| **Overview** \| **Package** \| **Class** \| **Tree** \| **Deprecated** \| **Index** \| **Help** \| **Weka's home** | |  |
| **PREV CLASS**   **NEXT CLASS** | **FRAMES**    **NO FRAMES**     **All Classes** |  |
| SUMMARY: NESTED \| FIELD \| CONSTR \| METHOD | DETAIL: FIELD \| CONSTR \| METHOD |  |


---
